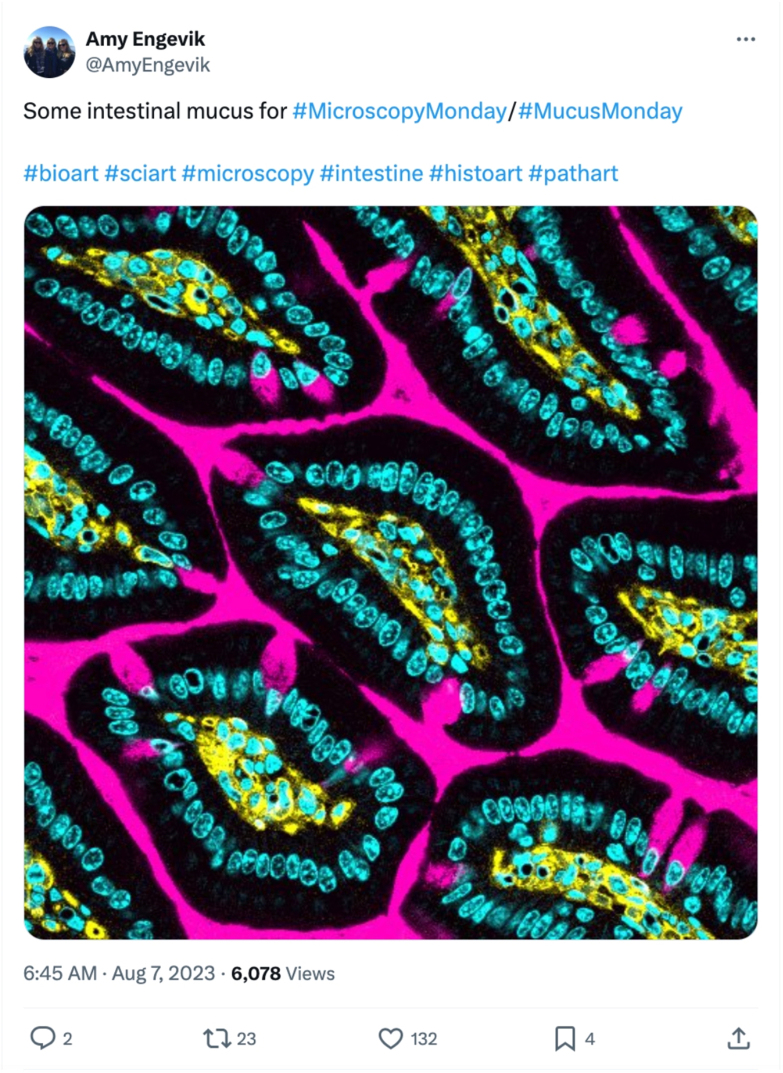# #PathArt: from glass slide to canvas; with a mission of enlightening the burdens of life

**DOI:** 10.1016/j.acpath.2024.100157

**Published:** 2025-02-03

**Authors:** Meredith Herman, Casey Schukow, Alexandra Tatarian, Ziad M. El-Zaatari, Gloria Hopkins Sura, Marilyn M. Bui

**Affiliations:** a Department of Pathology, University of Michigan, Ann Arbor, MI, USA; b Department of Pathology, Corewell Health, Royal Oak, MI, USA; c Department of Undergraduate Medical Education, State University of New York Upstate Medical University, Syracuse, NY, USA; d Department of Pathology and Genomic Medicine, Houston Methodist Hospital, Houston, TX, USA; e Department of Pathology, Moffitt Cancer Center & Research Institute, Tampa, FL, USA

**Keywords:** Art, History, Medical art, #PathArt, Pathology, Social media, Twitter, X

## Abstract

Pathology requires visual aptitude, pattern, and color recognition as a medical specialty. This can account for the growing PathArt (or #PathArt via social media, or SoMe) movement. For the purpose of this review, the authors define PathArt as any form of art inspired by pathology topics, such as microscopic images (i.e. surgical histology, cytology, hematology, immunohistochemistry), gross pathology, and clinical pathology (including molecular/genetics). Pathologists are well-versed in the use of hashtags and commonly utilize them to tag relevant medical topics to share with colleagues through online platforms, such as Twitter (renamed X in 2023). As the professional laboratory network has expanded virtually, artists within the community have emerged and shared numerous pathology artworks. However, displaying pathology as “beautiful” art pieces gives rise to concerns over portraying cancer light-heartedly given the humanity of disease. For this review, we discuss the history of art and medicine, pathology as a visual and creative specialty, explore the conceptual framework of the hashtag #PathArt is associated with sharing pathology-related art on SoMe, and address the psychological and medico-legal implications that surround PathArt. This article is intended to provide a guide to fostering PathArt and #PathArt in an ethical and positive manner. References were obtained via qualitative review of non-peer-reviewed and peer-reviewed literature pertinent to this topic.

## A brief history of art in pathology and laboratory medicine

The connection between art and pathology dates back as early as 1665 when Robert Hooke's *Micrographia* was published by the Royal Society.^1^ He is noted as the first physician to describe cells underneath a microscope and illustrate his findings. Hooke accomplished his illustrations with one eye looking through the microscope and the other eye on a sheet of paper, where he sketched what he was visualizing. His work was inspired by Antoni van Leeuwenhoek in the 1670s; however, Leeuwenhoek's drawings have been described as “crude” compared to Hooke's elaborate sketches. Interestingly, his simplistic approach also helped him to compare the magnification of his different instruments, suggesting he had substantial practice in refining the precision of his sketches. His hand-drawn sketches were then engraved onto copper plates for printing and distribution. Comparison between the visual and written forms of communication between these two early scientists demonstrates how their unique talents powerfully conveyed the earliest documented microscopic observations.

Later in the nineteenth century, some doctors who traversed both medical and artistic careers were known as talented “draughtsmen” using observational and illustrative talents to capture their work without formal training.^2^ Examples of such individuals include Jean-Martin Charcot and Paul Richer (who sketched, modeled, and sculpted) and Charles Bell (who etched illustrations of his dissections into publications for medical students). Since medicine and the arts both involved intense training and professional devotion, some doctors left medicine to become full-time artists, while others focused on scientific discovery and medical practice.

Scientific illustrations are not new to medicine. Scientists have gleaned from original illustrations to understand cellular intricacies even before photography and advanced photo-capturing technology existed. Japanese wildlife artist, Hashime Murayama, worked for National Geographic as an illustrator in the 1920s until his friend, George Papanicolaou, asked for help to illustrate cervical cancer cells.^3^ This collaboration showed the importance of art in conveying scientific findings, particularly in the discovery of pre-cancer screening in Papanicolaou (Pap) smears.^4^ Several decades later, a renowned 20th-century textbook written and illustrated by Dr. Richard DeMay, *The Art and Science of Cytopathology*, exemplified the necessity for imagery in the field of pathology.^5^ Published originally in 1996 (now a multi-volume encyclopedia set), DeMay included a highly detailed, colored atlas of thousands of pathology images, tables, and illustrations to describe cell types, morphologies, stains, and application to differential diagnostic processes.

Through technological and medical advancement, pathology has now shifted away from hard-copy illustrations and towards the digitization of images. Nevertheless, the historical importance of illustrations to share scientific findings, particularly microscopic images, must be appreciated in this review of pathology art. This article explores the crucial connections between medicine (specifically pathology) and the arts (i.e. PathArt), identifies the community around this artistic phenomenon, and reviews the potential positive benefits and medicolegal implications of PathArt (and its hashtag SoMe counterpart, #PathArt). The intention of this article is not to provide an exhaustive systematic or scoping review, but rather a qualitative and semi-quantitative narrative of current non-peer-reviewed and peer-reviewed literature pertinent to this topic as determined by the authors.

## PathArt in art and medicine

To understand PathArt and its role in art and medicine, we should first understand the broader context of art in medicine. Historically, art involved detailed drawings, paintings, and models which were used to illustrate complicated scientific principles to the greater community.^6^^,^^7^ In the 21st century, art became more than a tool to educate scientists of their findings, but was integrated into holistic medicine via caring for the “whole person”.^8^^,^^9^

Various forms of art have since been used to facilitate a connection between healthcare professionals and their patients across diverse cultures, spiritual and religious backgrounds, personal and societal experiences, thoughts, and emotions.^10^, ^11^, ^12^ This connection has led to more empathic care for each patient. Integrating the arts in medicine also contributes to improved healthcare outcomes, patient and staff satisfaction, and lower healthcare costs.^13^ Moreover, art can be used to help patients better understand their illness. One free resource, known as MyPathologyReports.ca, was created to help patients comprehend their pathology reports through simplistic language and medical illustration in one accessible website.^14^ As a result of these efforts, patients have become more empowered as advocates for their health.

The act of creating art can also bring patients of all ages and caregivers alike happiness, wellness, a sense of community, comfort in the face of illness, and improved quality of life.^9^^,^^15^ Also, art integrated into medical education (MedEd), as evidenced by an increasing number of recent studies^16^ can facilitate learning the skill of empathy, objective and subjective observational skills, communication skills^17^ and tolerance for ambiguity.^18^

Based on the current published literature, PathArt fulfills at least two major roles of art in medicine: (1) connecting with patients as “a whole”, (2) wellness from creating and sharing art, and (3) medical education. Perhaps nowhere else is a better demonstration of how art can connect patients and their pathologists as in Dr. Marilyn Bui's book *The Healing Art of Pathology*,^19^ in which patients and pathologists share works of art that reflect on each of their experiences on either side of a pathologist's diagnosis. In the book, artist and cancer patient Ray Paul combines histologic images of his own tumor cells with paint and other techniques to make art as “a persistent, visual manifestation of the battle raging within and a powerful testament to the beauty of hope”.^19^ Also in Bui's book, pathologist Dr. Shahla Masood reflects on her painting called “Praying Child” and “how a pathology report with sad news can initiate a similar image of praying for a possible miracle”.^19^ Pathologists as artists is not new to the field. Known pathologists have also sought additional training in medical illustration to further combine their medical training and artistic inclinations.^20^

For the role of PathArt in MedEd, Carcolici et al.^21^ recently conducted a pilot study in which medical students created visual arts as a tool to help in histology education. Results showed that the majority of participating students viewed art as a valuable resource for learning medical concepts. This is not surprising given the inherently visual nature of surgical pathology and histology. Moreover, three decades prior, then-medical students Peter A. Gearhart and William J. Nicholson commented that views under the microscope “captivate the imagination of the viewer, inspire an increased recognition of patterns, and greatly enhance learning”.^22^ Practicing pathology has been likened to an art form, as Dr. Saul Suster writes “watching him [a chief pathologist] make a diagnosis was like watching a painter paint his masterpiece or a composer create a haunting melody”.^23^ Suster describes the process of arriving at a diagnosis in difficult cases in surgical pathology as a highly creative one in which multiple pieces of information and minute clues are gathered and finally crafted into an interpretation. Thus, inherent in the education of every pathologist is the education of a PathArtist, and even some of the best artists look at medicine for inspiration.^23^

## PathArt compared to the arts of surgery and radiology

The work of surgeons, pathologists, and radiologists depends on strong connections between the eye and mind to achieve precise and accurate visualizations and subsequently well-founded judgments informing key aspects of patient care. This begs the question, *are other non-pathology specialties artistically inclined?* Most healthcare professionals are surprised to learn that the field of art and the field of medicine historically share the same patron saint, St. Luke.^24^ Anyone who understands both fields intuitively knows that they also share a certain curiosity, a desire to change reality, and a mission to lighten our burdens of life.

An article by Dr. Steven Neal, who describes himself as becoming an artist before a physician,^25^ allows for a comparison between the art of plastic surgery and pathology. He first puts forth that medical school training is based on left-brain skills, so many residents lack the natural ability and proficiency in their early attempts at plastic surgery, a right-brained endeavor. Furthermore, he states that plastic surgeons who achieve superior results possess judgment, a skill that cannot be learned through conventional technique. Pathology is also a crossroads for the left and right brain since there is a strong visual component in determining a diagnosis.^26^ Experience over time leads to the development of diagnostic expertise and sharpness of the eye, which are important in a field where finding one distinct cell can make a life-changing difference.

A surgeon's judgment involves their ability to make decisions based on observations made with the eye, including the size of modifications during a procedure and how variations should be managed. This practice is comparable to pathology, where visual observations of cell size, appearance, and number, especially in comparison to other cells, inform the resultant diagnosis. Neal shared that in working with hundreds of surgeons,^25^ he has found a large variety in the ability to judge with the eye and in the judgments made by practicing physicians. This aspect is also similar to pathology, since judgments, such as staining positivity and percent cellularity of bone marrow, can vary slightly between pathologists and institutions. Consequently, there are regular calibration exercises and laboratory accreditations with national standards in place. Overall, accurate judgment with the physician's eye is important in both pathology^27^ and plastic surgery, because improper judgment can result in an undesired change in appearance, or misdiagnosis, respectively.

Three-dimensional practice is another artistic element common to both surgery and pathology. Neal teaches that a trainee does not know the nose in three dimensions until they can sculpt it from memory, which involves translating memory into reality.^25^ Pathologists also require refined spatial visualization skills and must possess a strong memory of patterns, microscopic appearances, and anatomy. For example, in processing surgical excisions for frozen sections and gross anatomical evaluation, the ability to orient specimens in the body and accomplish adequate depth in sampling is critical, especially in reading margins for cancer cases. One could argue that these skills easily translate to artistic applications.

While surgeons and pathologists are linked by specimen and anatomy, pathologists and radiologists are connected by the nature of their observation-based diagnostic work. Pathologists look under the microscope and review slides in color, while radiologists typically interpret images in shades of gray, both with the task of describing visuals to provide critical data to clinicians that will inform patient care.

The intersection of PathArt has been relatively unexplored in comparison to medicine as a whole ^28^^,^^29^ (hence, the purpose of this review). A study evaluating the link between radiology and fine art found that radiology is becoming a prominent field of modern art, not only because of the colors, compositions, and quality but also the symbolic, metaphorical, emotional, and contextual components of the images. ^30^ Radiology is a form of photography, and there have been many instances of radiologists creating art and artists creating radiographic works displaying parts of the body. While the work of a pathologist is similarly visual, there are likely more instances of radiology translating to art given the level of organization. For example, there is likely greater familiarity among the general population with radiographic images of bones and organs such as the heart and lungs, in comparison to the tissues, cells, and other microscopic life examined by pathologists. Also, there would be more accessible meaning deduced by the untrained eye from a radiograph of a broken bone in comparison to a specimen displaying microscopic dysplasia; thus, making pathology possibly less compelling than radiology for translation into art.

## What is #PathArt?

The professional utilization of social media (SoMe) platforms within pathology,^31^ particularly Twitter/X,^32^ has proven to be an efficient and effective tool for networking and collaborating across all ages and levels of training.^33^ Twitter (parent company: X Corp.) can be accessed through a free, downloadable app on cellular devices or through their website (www.twitter.com). Mukhopadhyay et al.^34^ highlight recommendations and best-practice guidelines for creating professional, engaging Twitter accounts. Additional SoMe platforms that are commonly used by the online pathology community include Instagram,^35^ Facebook ^36^ (parent company for both: Meta Platforms, Inc.), and KiKo (parent company: KiKo, LLC).^37^ Many of these platforms use similar hashtags for content related to this review (i.e. #pathology, #art).^38^

Hashtags have been shown to be an effective tool to categorize ideas or topics in a post, which is further populated into the SoMe platform and searchable in the app, and it also further targets a specific audience. In 2015, for instance, the hashtag “#PathArt” originated in a Twitter post (“tweet”) by Dr. Jerad M. Gardner. The pathology community quickly caught onto the movement and has since used this hashtag with posts related to pathology art.^39^ #PathArt now represents any form of art inspired by pathology topics, including gross and microscopic images, histology, cellular preparations, and scientific instrumentation.^40^ Other popular hashtags have emerged within the community, for example, #PathTwitter/#PathX tags an audience within the professional pathology and associated laboratory professions community. Other subspecialties in pathology have their own established hashtags, such as #hemepath for hematopathologists,^41^ to share specialty-specific knowledge virtually. SoMe tweets and their hashtags have multi-functional educational purposes and can boost professional conference engagement and excitement as well.^41^

To highlight some examples of tweets, a search of “#PathArt” on June 17, 2023, was performed on Twitter, which revealed top content and highest impressions (see Supplemental Figs. 1, 2, 3, 4, and 5).^42^ Select examples of how #PathArt by several of the authors of this review illustrates the dual purpose of showcasing pathology-related SoMe content while also providing MedEd are shown in Fig. 1, Fig. 2, Fig. 3.^43^, ^44^, ^45^

**Fig. 1:**
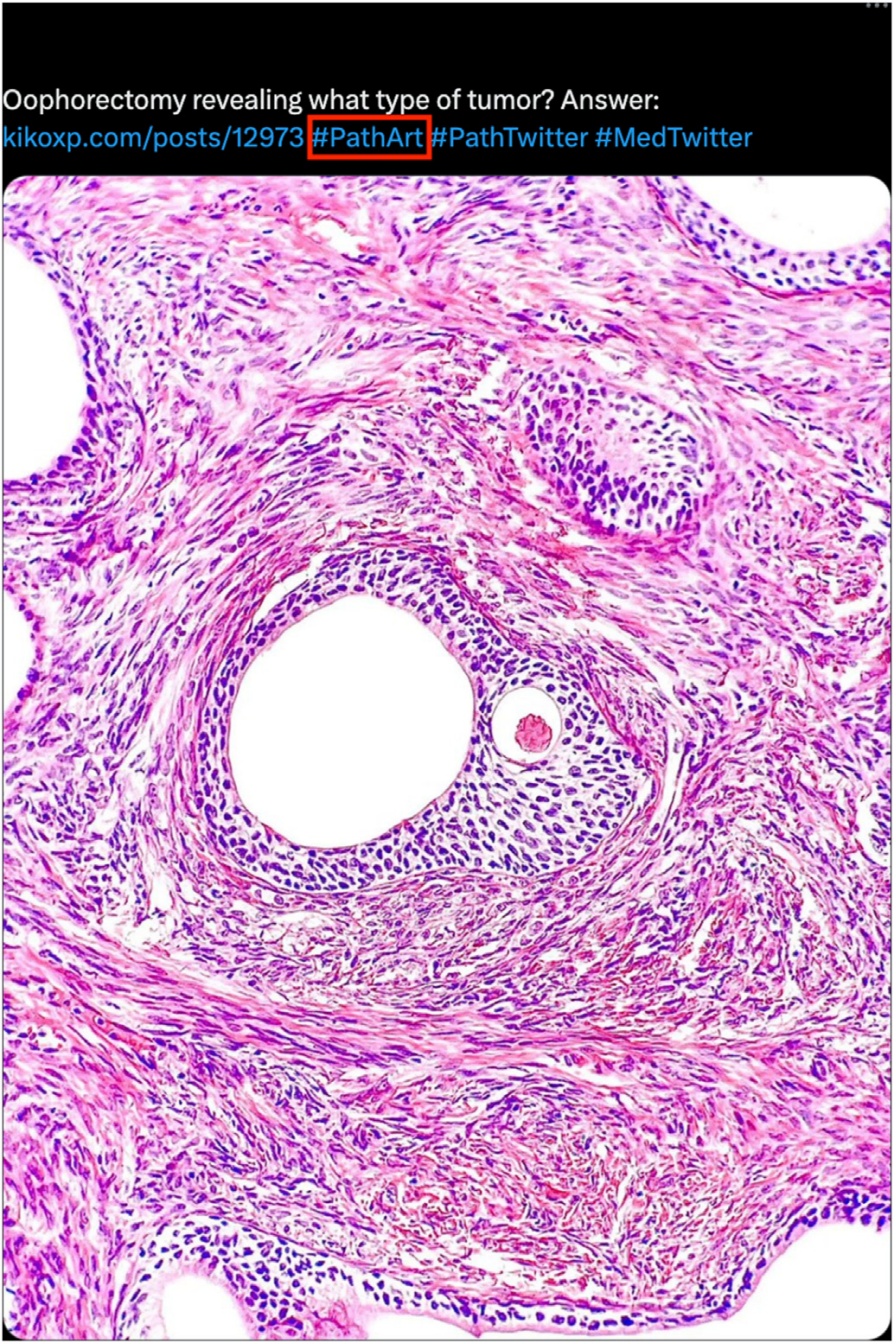
An example Twitter/X #PathArt (see red box) post by one of the co-authors (GS). In this figure, the author includes a MedEd question regarding an oophorectomy revealing a certain type of tumor while including an external KiKo link that readers can follow for answers and more information. Other relevant hashtags are included (i.e. #PathTwitter, #PathX, #MedTwitter). Relevant impressions (i.e. Twitter user engagement) as of June 16, 2023, include at least 12,900 views, 21 retweets, 1 quote tweet, 130 likes, and 9 bookmarks.^43^ Abbreviations: KiKo, Knowledge In Knowledge Out; MedEd, medical education.

**Fig. 2:**
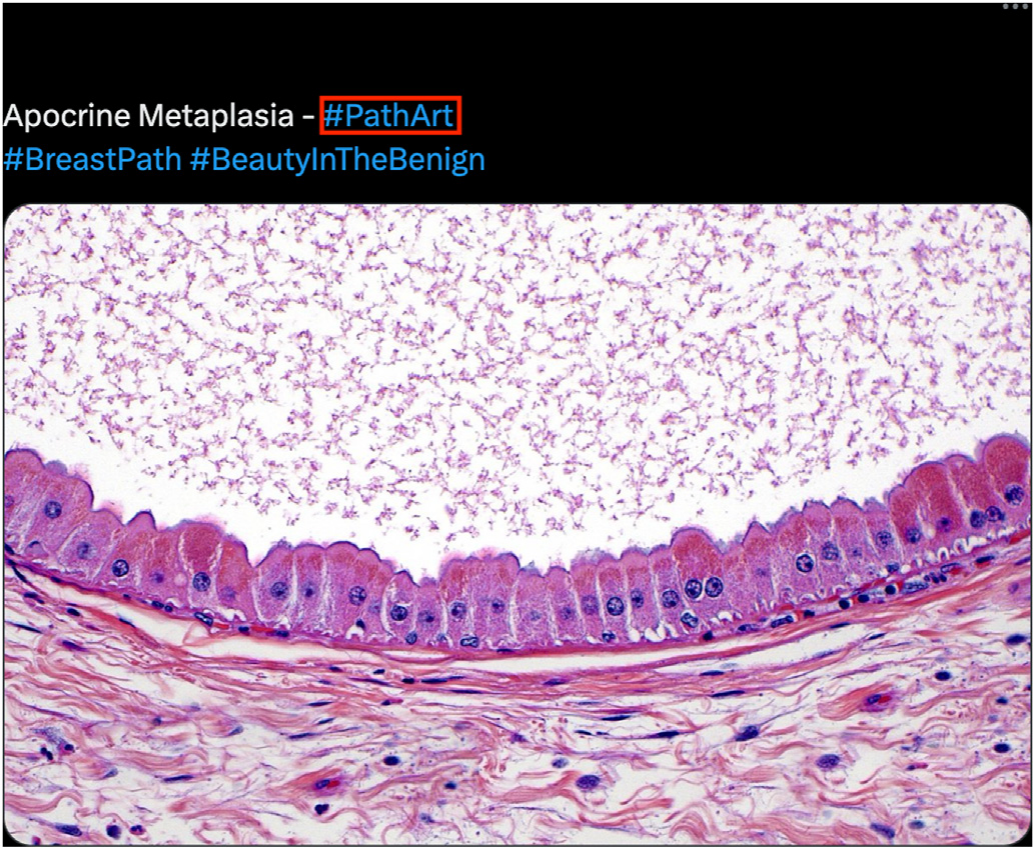
An example Twitter/X #PathArt (see red box) post by one of the co-authors (ZMZ). In this figure, the author incorporates #PathArt to showcase the breast pathology of apocrine metaplasia, while including other relevant hashtags (#BreastPath and #BeautyinBenign. Relevant impressions (i.e. Twitter/X user engagement) as of June 16, 2023, include 2,498 views, 11 retweets, and 73 likes.^44^

**Fig. 3:**
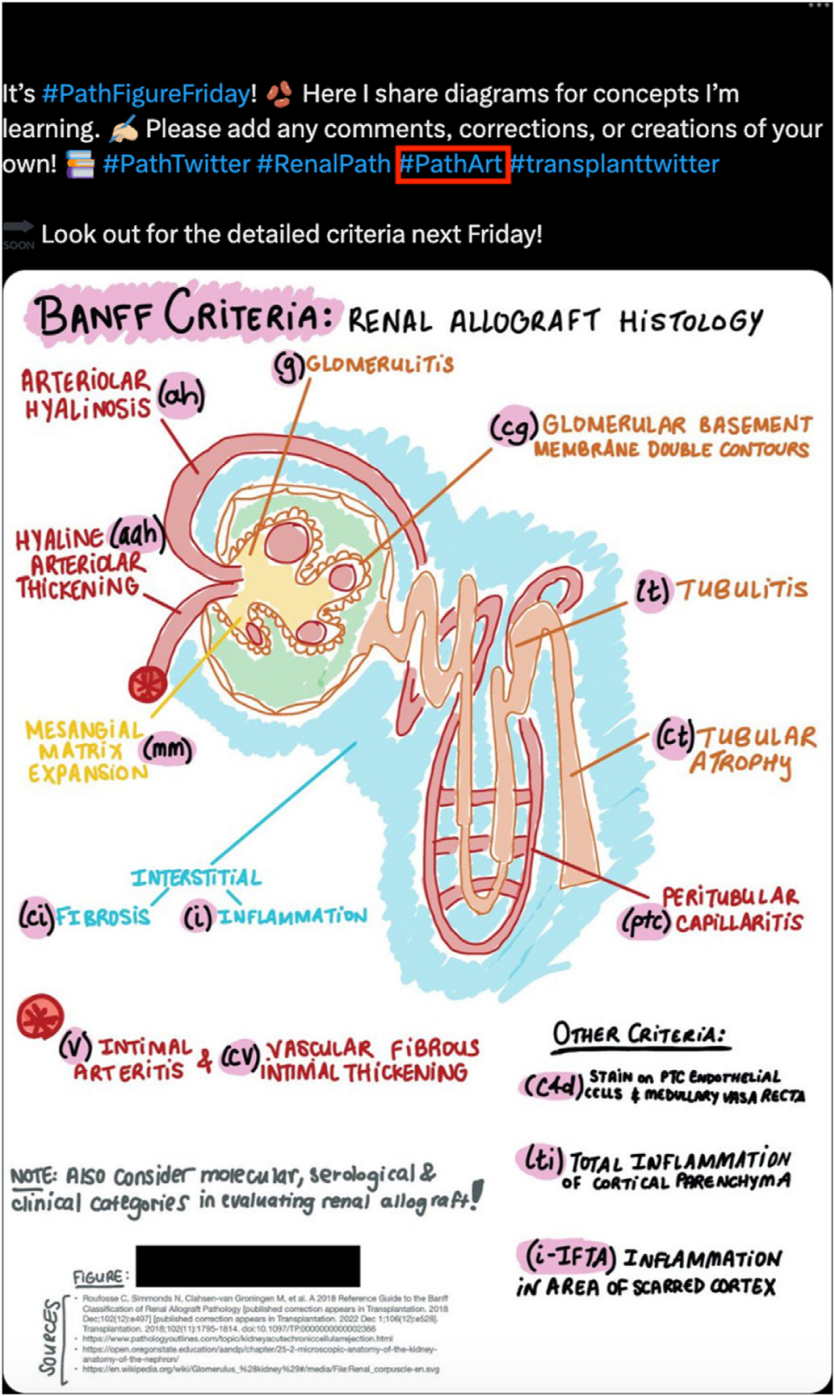
An example Twitter/X #PathArt (see red box) post by one of the co-authors (AT). In this figure, the author utilizes a hand-drawn diagram using the Notability app on her iPad to illustrate Banff Criteria and renal histology (denoted by relevant hashtags of #RenalPath, #PathTwitter, and #transplanttwitter). This author also incorporates appropriate emojis to give extra substance to her tweet, including kidney beans (representing kidneys), hand drawing, books, and arrows. The hashtag #PathFigureFriday suggests that this author uniquely releases similar hand-drawn MedEd pathology figures on social media on Fridays. Other relevant hashtags are included (i.e. #PathTwitter, #MedTwitter). Relevant impressions (i.e. Twitter/X user engagement) as of June 16, 2023, include 2,839 views, 9 retweets, 39 likes, and 4 bookmarks.^45^ Abbreviations: MedEd, medical education.

A brief search of #PathArt social media statistics was conducted on BrandMentions.com ^46^ from February 5, 2024 to March 3, 2024. The number of social media posts that used the hashtag #PathArt is shown in Fig. 4.^46^ Further breakdown of the number of posts in the top four most utilized social media platforms is illustrated in Fig. 5.^46^ Additional statistics on the cumulative performance of #PathArt posts are shown in Fig. 6.^46^ The reach of posts using #PathArt shows the total number of accounts reached on social media. As the algorithm shows content to users, a certain amount of them will interact with the post. An interaction is equivalent to viewing it for a period of time rather than scrolling past it, engaging with it via a like, share, save, or more. Within a 30-day time frame, 503,900 users saw #PathArt-related posts, with a smaller fraction of interactions as the posts reached their target audience, which totaled 20,700 likes and 18,100 shares. Further breakdown of #PathArt impact can be seen in Fig. 7.^46^ A list of notable social media users who consistently post PathArt content is shown in Supplemental Fig. 6.^46^^,^^47^ The value of social media cannot be underestimated as a tool to promote, share, and disperse content.

**Fig. 4:**
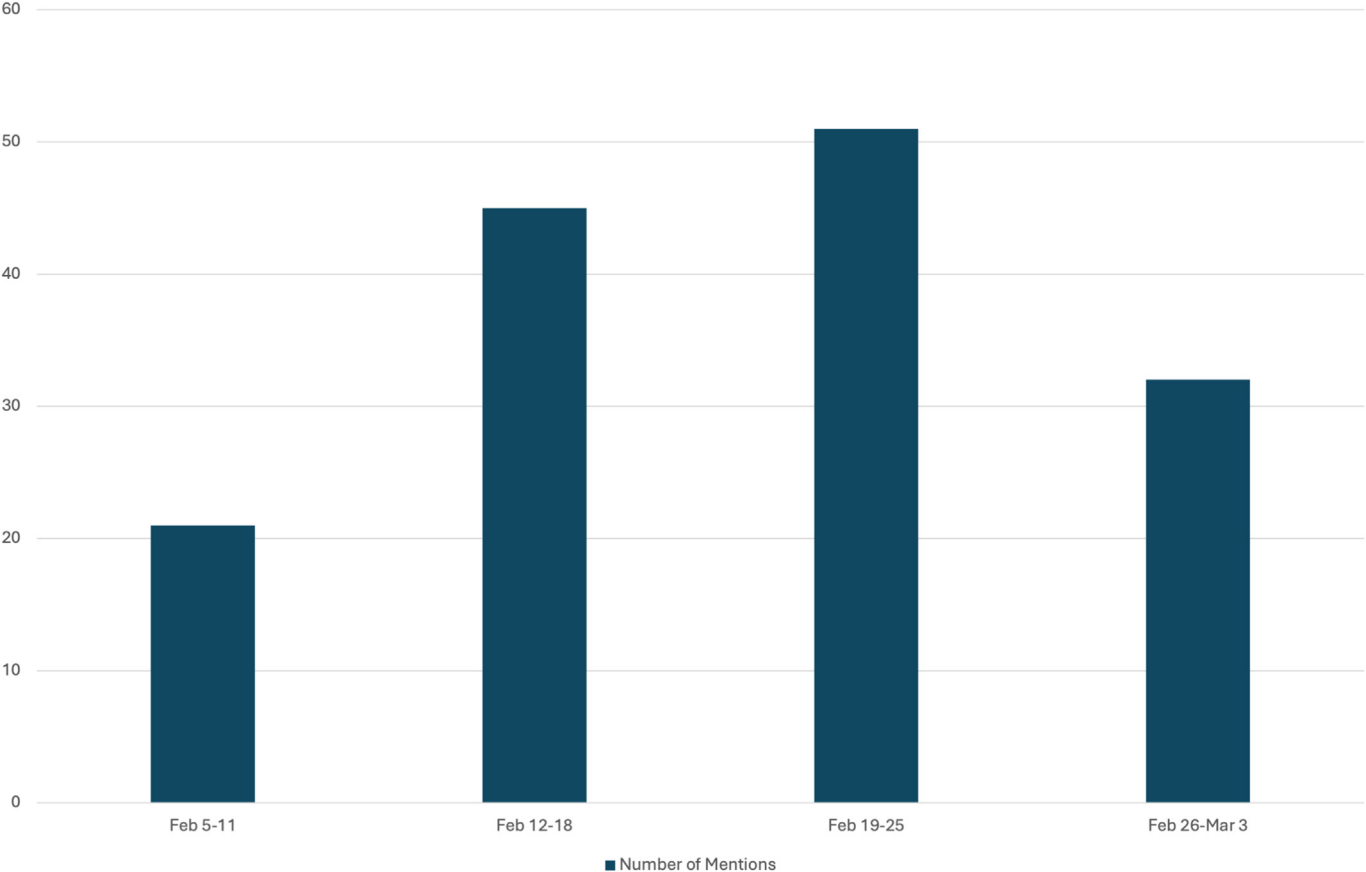
Cumulative impact (number of accounts reached and total views) when #PathArt was used on all platforms during each week from Feb 5 to March 3, 2024. Data from BrandMentions.com.^46^

**Fig. 5:**
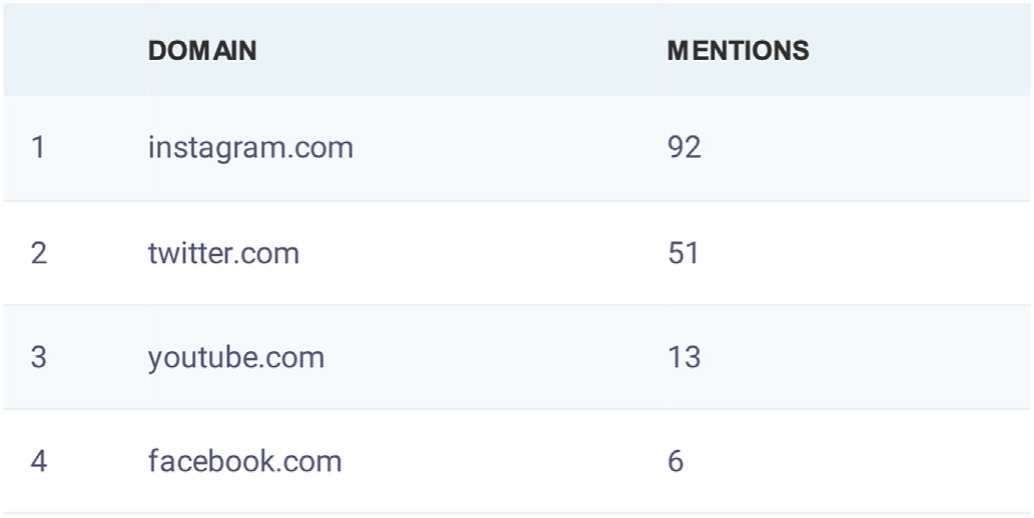
Mention history of the hashtag #PathArt on all social media platforms between February 5th to March 3rd, 2024. The number of mentions per week is shown in each bar. Data derived from BrandMentions.com.^46^

**Fig. 6:**
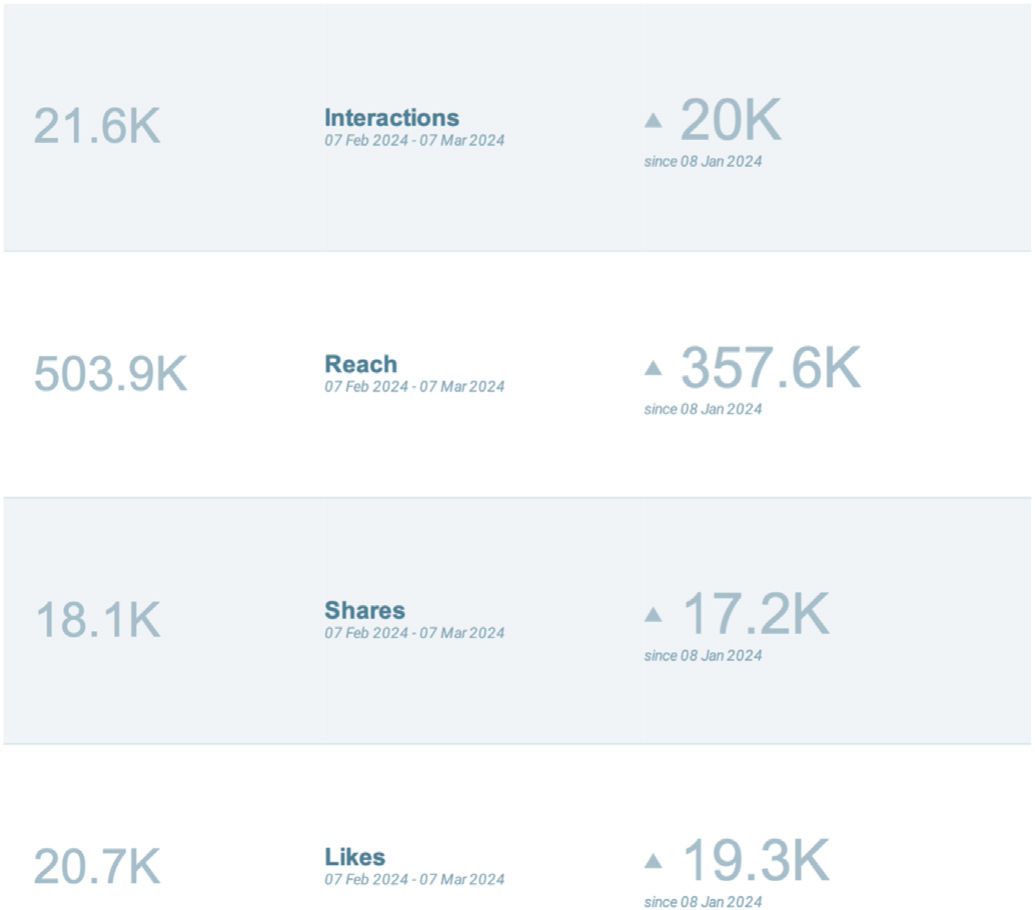
Top 4 social media platforms where #PathArt was utilized between February 5th to March 3rd, 2024. #PathArt is more frequently mentioned on Instagram and Twitter/X. Data derived from BrandMentions.com.^46^

**Fig. 7:**
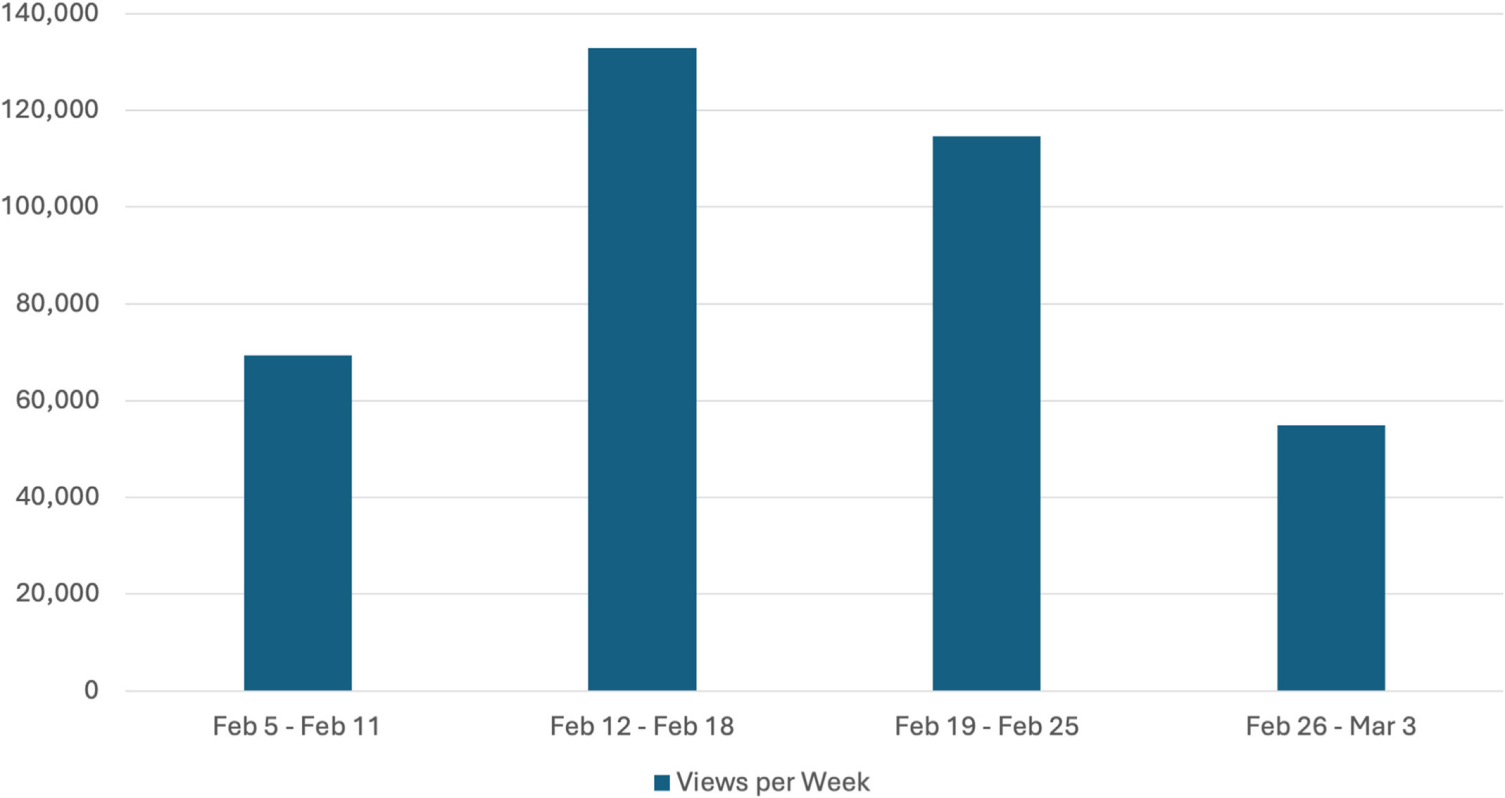
Metrics on the changes in #PathArt performance on all social media platforms compared to one month prior. Values in the left column indicate metrics in each area from Feb 5 to March 3, 2024. Values on the right indicate metric performance from the month prior. This indicates the use of #PathArt is growing in all domains. Data from BrandMentions.com.^46^

Outside of social media, the use of “art” in medicine is a familiar term, yet it takes on different meanings. In non-pathology specialties, there is art in clinical decision-making and documentation,^48^ suturing and surgical techniques,^49^ and radiology imagery. ^50^^,^^51^ (see “A comparison of the art of pathology with the arts of surgery and radiology” section for more information). Baruch ^48^ states that creativity is an essential component of being a physician and that “our worldview informs what we notice and how it is interpreted … the product represents an extension of our minds and our intentions outside ourselves”. Both the microscopic images and educational illustrations of pathology themes have further expanded into creative and inspirational purposes. The visual nature of diagnostic specialties, such as pathology, can account for the growing #PathArt movement and utilization of hashtags on SoMe.

The concept of creativity aids in performing duties required of physicians to apply medical knowledge to complex clinical scenarios. The view of doctors as “makers”^48^ further conveys that despite the accessibility to medical knowledge via the internet and SoMe, medicine is still everchanging and “bottomless”, requiring doctors to inquire into what is known and not known. “Not knowing is an openness of mind for considering complex or ambiguous cases”, Baruch further writes, and not knowing thus forces doctors to form an open-mindedness and form connections between various ideas. Such complex thought and creative skills lead to innovation through “critical thought, cognitive flexibility, and learning agility”,^48^ which transcends these ideals and definitions of art in medicine and PathArt/#PathArt.

## PathArtists

PathArtists, in the context of PathArt, broadly are individuals who create and share a finished product on SoMe (often denoted by #PathArt) or other platforms whilst including information about pathology. Although PathArtists do not necessarily need to be active on SoMe to still create PathArt, SoMe versus other virtual online modalities allows artists to freely distribute their name, likeness, and materials to a wider global audience. They may be self-taught medical professionals or trainees who create artworks based on imagery seen underneath the microscope or art inspired by experiences within the laboratory. Or, as in the case of Roy mentioned earlier,^19^ could be artists at baseline who are inspired by medical phenomena. Regardless, such artists have emerged through SoMe and for the purposes of this review, were referenced after performing a Twitter search of “#PathArt” on June 6, 2023.^42^

**Fig. 8:**
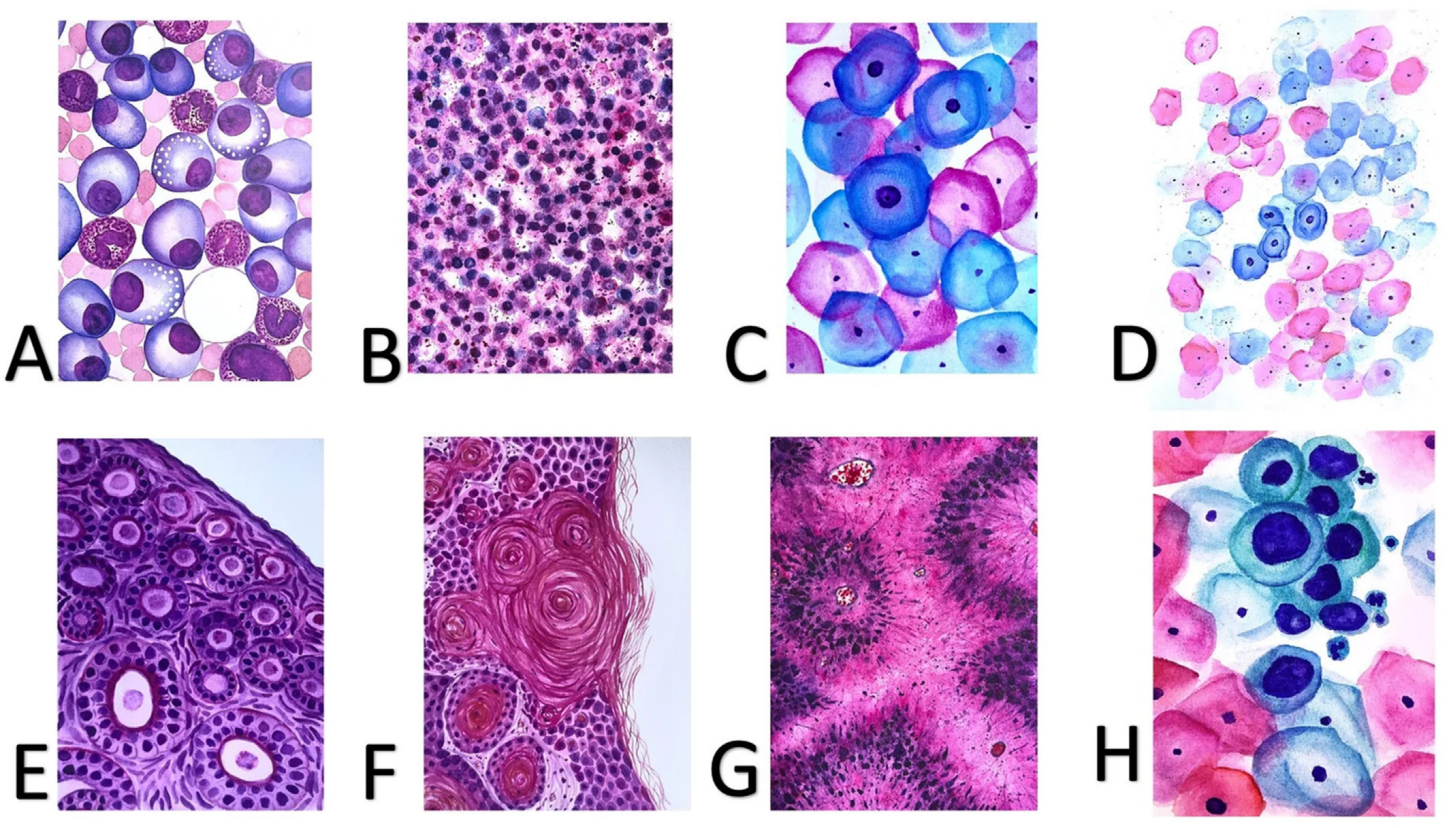
A series of 8 blank cards with hand-painted PathArt (A–H) from White Coat Artistry. Permission was obtained for its use in this review by Meredith Herman (MH). Please refer to Table 1 for more information regarding White Coat Artistry.

PathArt may take the forms of paintings, digital artwork, diagrams, photographs, paper art, embroidery, woodworking, jewelry, etc. #PathArt goes beyond sharing art pieces virtually, too. One of the authors of this review (MH) is a PathArtist who has taken her pathology training and artistic talents to TikTok^52^ and other SoMe platforms to showcase visually appealing beauty of life under the microscope (see Table 1 and Fig. 8).

**Table 1:** Freely accessible online platforms for White Coat Artistry with owner names, SoMe handles, and hashtags where applicable.

TikTok Meredith Herman, DO (@meredithkherman); search #whitecoatartistry
*Twitter* Meredith Herman, DO (@MeredithKHerman); search #WhiteCoatArtistry
*Instagram* Meredith Herman (@whitecoatartistry); search #whitecoatartistry
*Facebook page* White Coat Artistry; search #whitecoatartistry
*Etsy* WhiteCoatArtistry

Abbreviations: SoMe, social media.

Other ParthArtists have created art businesses by creating and selling original prints of their art, too. Most pieces demonstrate some knowledge regarding the gross or microscopic intricacies of the human body, and the authors encourage readers to explore their works for purchase from online marketplaces such as Etsy.com and Amazon.com after searching keywords “pathology art”. While the authors recognize that more research is required to properly evaluate the intricacies and impact behind PathArt and PathArtistry in pathology, this discussion will hopefully provide a nidus for further studies.

## PathArt competitions

Competitions in pathology art have become popular in recent years and have encouraged the broader pathology community to create and share their artistic creations. Individual pathology departments and residency programs hold internal PathArt competitions and were identified from responses to a tweet by one of the authors (ZMZ) on June 4, 2023 (available here: https://twitter.com/ziad_zaatari/status/1665489873866727424). Departments with PathArt competitions identified via this tweet included Mayo Clinic (@MayoClinicPath), Baylor College of Medicine (@BCM_PathRes), Brigham and Women's Hospital (@BWHPath), University of Iowa (@UIPathology), and Washington University (@wusm_pathology).

Albeit this is not a complete, nor exhaustive list, as the searching methodology was informal without a stepwise approach or searching through other SoMe platforms or via relevant pathology organization listservs (e.g. College of American Pathologists, American Society for Clinical Pathology, United States and Canadian Academy of Pathology, British Association for Cytopathology). Through a qualitative review of “pathology art competition” Google search in June 2023 and anecdotal word-of-mouth, the authors have composed a list of other institutions that have held pathology art competitions open to the public or the broader pathology community with links provided where appropriate (see Table 2).

**Table 2:** Institutions that have held PathArt competitions open to the public or the broader pathology community with links provided.^a^

Turkish Journal of Pathology http://www.turkjpath.org/contest_2022.php?id=81
*College of American Pathologists Resident's Forum* https://documents.cap.org/documents/2017-fall-residents-forum-meeting-photo-contest-results.pdf
*The Royal College of Pathologists* https://www.rcpath.org/discover-pathology/competitions/art-of-pathology-competition.html
*American Society of Cytopathology* https://cytopathology.org/page/PhotoContest
*Arkana Laboratories* https://www.arkanalabs.com/2022-art-of-medicine-competition/
*American Society for Microbiology* https://asm.org/Events/ASM-Agar-Art-Contest/Home
*American Association of Pathologists' Assistants* https://www.pathassist.org/news/605575/The-AAPA-Photo-Contest-will-be-gone-in-the-blink-of-an-eye-.htm
*International Federation of Clinical Chemistry and Laboratory Medicine* https://ifcc.org/about/ifcc-celebrates-70-years/art-competition/
*Fedpatmex: The Mexican Federation of Anatomic Pathology* https://fedpatmex.org/
*Dunn School of Pathology* https://oxsci.org/dunn-school-of-pathology-art-competition-a-review/
*University of Toronto* https://lmp.utoronto.ca/art-competition
*Mosul Medical College* https://uomosul.edu.iq/en/medicine/the-department-of-pathology-announces-the-results-of-the-fourth-pathology-arts-competition/
*Pakistan Association of Pathologists* http://pakpathology.org/2020/07/18/the-art-of-pathology-competition-is-now-open-to-uk-and-international-entrants/
*British Association for Cytopathology* https://www.britishcytology.org.uk/image-of-the-month

Abbreviations: PathArt, pathology art.

a As of June 2023.

A notable international photomicrography competition not restricted to pathology but certainly garnering participation from pathologists since its founding in 1975 is Nikon's Small World photo contest as well (https://www.nikonsmallworld.com/).

## Literature and exhibits on PathArt

Bui and Galagan's 2016 *The Healing Art of Pathology*^19^ (as mentioned earlier) recognizes the “centuries-old, [l]argely beautiful, [s]teeped in history, and [g]rounded in the practical'' concepts which pathology (whether benign or malignant disease) and art are. As to “[w]hy an art book in pathology”, Bui and Galagan state:^19^

*I thought of my [Bui] patient, Ray, an artist who turned photomicrographs of his tumor into powerful art after we reviewed his slides under the microscope together. Being a small part of his courageous journey to understand and fight his cancer has truly inspired me. Ray serves as a constant reminder that behind every case there is a deserving human spirit that commands the best from me as a pathologist, educator, researcher, and advocate for our profession.*

Per Ray, who was diagnosed with sarcoma in 2011, he connected with Bui (the pathologist who helped diagnose his cancer) and wanted to see what his tumor looked like under the microscope (staring the “devil in the eye”, so to speak).^53^ Moreover, a collaboration between Roche Tissue Diagnostics, the Southern Arizona Arts and Cultural Alliance (SAACA), and Dr. Bui showcased pieces from Ray Paul and Dr. Mary Lachman in 2019.^54^

This collaboration has inspired others in the field to share their takes on and dedicate publications towards PathArt. The same year their book was published, *The Pathologist* magazine shared an article on the power of PathArt in SoMe via its same-name, community-building #PathArt hashtag,^55^ including a separate article on Bui and Galagan's mission of “art from the heart”.^56^ Since then, this widely-viewed magazine has released an annual image and multimedia art galleries (e.g. immunostains, hand-drawn illustrations, baked goods) submitted by pathologists, trainees, students, and other laboratory professionals.^57^, ^58^, ^59^ In 2018, Fan,^60^ a general surgical pathologist, also released a book of personal sketches to shed light on the complexities within bone and soft tissue pathology, offering his interpretations and observations through a series of cases.

Admiration for pathology art has also expanded to include art exhibitions and museums around the world. The Vienna Pathological-Anatomical Museum, housed within the Old General Hospital, also known as The Fool's Tower, gives visitors an inside look into a collection of 4000 pathological specimens.^61^ In Berlin, Germany, the Berlin Museum of Medical History at the Charité exhibits a permanent collection called “On the Trace of Life” which gives visitors an inside look into the evolution of medicine and leads them to the specimen hall, a collection of 750 pathologic specimens owned by the renowned German Pathologist, Rudolf Virchow.^62^

In Indianapolis, Indiana, the Indiana Medical History Museum within the Old Pathology Building is one of few remnants of the demolished Central State Hospital. Since 1896, this building has served as a place for physicians to study mental diseases as evidenced by the extensive collection of tools used in autopsy, a large library, histology room, and preserved brain specimens. To preserve the stories of patients who received treatment at this mental hospital, the museum launched a program called “Voices from Central State” to bring humanity to the diseased brains exhibited at the museum.^63^

In the modern-day era, pathology continues to be viewed as an art form. After conducting a brief, qualitative review of abstracts and titles on PubMed via a keyword search of “pathology” AND “art” in June 2023, publications regarding PathArt date back to at least the 1980s^64^, ^65^, ^66^, ^67^ and since then have spanned each decade. In 1997, Silverman^67^ recognized the importance of patterns in pathology as a case that pathology is just as much art as science, which was followed up by Gearhart and Nicholson in 1998.^22^ Around a decade later, Reed^68^ discussed the imposition of virtual images on the art of pathology, recognizing the arbitrary nature of the neoplastic spectrum when diagnosing malignancies. Coyne and Whooley^69^ further discerned images in pathology as art in 2009, too.

In the 2010s, publications on PathArt began showing updates on the matter, such as via computational pathology,^70^ basic histology,^71^ infectious diseases,^72^ and even references to famous painters (e.g. Modigliani)^73^ and pop culture icons (e.g. *The Simpsons*).^74^ Towards the end of the decade, Nocito and Berra^7^ in 2018, described a collection of around 100 pathology-depicting moulages (wax models) of various dermatologic diseases featured at the School of Medicine of the National University in Rosario, Argentina. Finally, in 2023, Suster^23^ argued that pathologists are artists in “suits” and that “having an artistically inclined brain becomes a singular asset” when interpreting surgical specimens and crafting unusual or difficult diagnoses.

## Ethical aspects of PathArt

“[V]isiblity is central to the shaping of political, medical, and socioeconomic decisions” according to a 2014 article by Pietrzak-Fanger and Holmes on disease, communication, and the ethics of (in)visibility in light of the 2013 Ebola outbreak.^75^ There has long been a difficult relationship between increasing the visibility of disease while maintaining medicolegal and ethical methods of doing so. Although artists have historically been able to shed light on the nature of human suffering through visual depiction of disease and its relationship to society, not every artist throughout history has kept to this same moral standard.^76^ Art's place in MedEd appears to have steadily gained notice over the past two decades, and Dalia et al.^17^ have found that incorporating art into MedEd has curricula and individual benefits. Despite the fact that more research needs to be done to determine which art methodologies are most likely to result in clinically significant improvements in visual perception skills, empathy, and tolerance of ambiguity,^17^^,^^76^, ^77^, ^78^ current studies support the continued integration of art and humanities in MedEd.^73^

Pertaining to PathArt, then, is it ethical to create or share the art of malignancy or human disease? Art is a form of expression, including expressions of human misfortune or suffering. 79, 80, 81 In medicine, it may allow for the translation of patients' unique lived experiences regarding their illnesses and translating them with the vantage points of the physicians who care for them.^10^^,^^12^^,^^81^^,^^82^ Furthermore, engaging in art by healthcare providers and patients reduces adverse physiological and psychological public health outcomes and opens the door for conversation and connection with respect to disease.^11^^,^^83^, ^84^, ^85^ The intent of art in pathology, thus, is to educate and raise awareness alongside or through the means of uncovering the visual beauty and appeal present in pathologists’ work while capturing human expression and with intention to “lighten our burdens of life”.^71^

A world-famous anatomical art exhibition in Las Vegas, Nevada, called “BODIES: The Exhibition” displays entire real human bodies and organs in a three-dimensional video.^86^ While cadavers are a common learning experience for medical professionals, such meticulous dissection and display of human bodies gives people a once-in-a-lifetime view inside the human. Bodyworlds is another, similar exhibition of plastinated anatomical specimens.^87^ While both are popular exhibits, the acquisition and preservation of human bodies do raise ethical concerns. From the authors’ understanding, neither the owner of the BODIES nor Bodyworlds have been able to provide proper documentation of voluntary consent for body donation.

In fact, it has been publicized that the cadavers in BODIES: The Exhibition are “unclaimed” bodies with no valid proof of ethical consent have been obtained, and the Laogai Research Foundation has stated that these unclaimed bodies “includes executed political prisoners”.^87^^,^^88^ However, some parts of the exhibit are “clearly educational”, barring ethical controversy between “informed, voluntary and capable consent” and providing MedEd.^89^ Furthermore, Bodyworlds has not provided publicly available ethical documentation either, which has been acknowledged in peer-review literature.^90^^,^^91^ For these and other legitimate medico-legal concerns involving the hosting of plastinated and dissected human cadavers for educational exhibits,^91^, ^92^, ^93^, ^94^, ^95^, ^96^, ^97^, ^98^, ^99^, ^100^, ^101^, ^102^, ^103^ there still exists bioethical hesitancies regarding this practice even though it may provide visitors with more extensive knowledge of human anatomy and pathology.^99^^,^^100^

From a microscopic point of view, creating (or re-creating) gross or histologic images of human disease must be done so while considering ethical codes of conduct that all pathologists must follow regarding patient health information (PHI).^104^ According to the Health Insurance Portability and Accountability Act of 1996 (HIPAA) Privacy Rule regarding PHI, “if health information does not identify an individual or if there is no reasonable basis to believe that the information can be used to identify an individual, the information is not considered individually identifiable health information by federal law”.^102^ Therefore, sharing de-identified pathology images (whether they are intended as PathArt) does not violate HIPAA, even if shared on SoMe.^103^, ^104^, ^105^, ^106^

There are etiquette considerations that pathologists should keep in mind when creating or engaging with others' PathArt. Even though histopathologic images of malignancy may be beautiful as they can be visually pleasing,^107^ pathologists must remember that PathArt depicts human disease and suffering. Hence, pathologists who do create PathArt should, above all else, do no harm with their work and promote patient well-being with neutral-yet-inspirational artistry.^106^ “Beauty”, as it is said to be in the eyes of the beholder, can be drawn from malignant images because of their diagnostic features and elements of art itself (i.e. pattern, color, textures). But, pathologists, trainees, and medical students who produce PathArt must tread with caution to use their art for expression, education,^21^ and connecting with patients without causing harm (i.e. describing the histology of sarcomas may be sources of inspiration for some patients but may be stressful for others). Broaching patients’ understanding of their diseases and gathering verbal consent regarding their want to learn more about histologic intricacies is essential. If these considerations can judiciously be followed by the pathology community, there should not be ethical contraindications to PathArt as long as the intent is not malevolent and patient privacy is protected.

## Conclusion

PathArt bridges the science of medicine exemplified by the field of pathology, and artistic expression in medicine more broadly. It has existed since the beginnings of pathology and has risen in popularity with the modern advent of SoMe platforms. PathArt reveals the intersection of art and gross/microscopic diagnostic medicine^108^ as created by a variety of active PathArtists and popularized by many current competitions, exhibitions, and publications.

Medicine is closely linked to art throughout history. From the sketches of the human body by DaVinci in the Renaissance era^109^ to the detailed illustrations of cervical cancer cells by Murayama, scientists and artists alike have sought to understand the intricacies of human life.

The change of art from the purpose of sharing information to art as a form of artistic expression may be attributed to the advancement of digital imaging and global sharing on SoMe platforms like Twitter/X (with #PathArt as a popular hashtag denoting PathArt content). As long as patient confidentiality, community, and bioethical standards are followed, PathArt will remain widely accepted as a crucial pillar in the visual diagnostic specialty as a pathway to enlighten the burdens of life.

## Disclaimer

Permission was obtained by all authors for using the images as figures, and the intention of displaying their tweets as figures is not to boost their SoMe exposure for more followers or notoriety, but rather to provide readers with examples of how to incorporate #PathArt within SoMe posts which they can reference for their own endeavors. Furthermore, authors’ names and Twitter handles were anonymized for peer review.

## Funding

The article processing fee for this article was funded by an Open Access Award given by the Society of ‘67, which supports the mission of the Association for Academic Pathology to produce the next generation of outstanding investigators and educational scholars in the field of pathology. This award helps to promote the publication of high-quality original scholarship in *Academic Pathology* by authors at an early stage of academic development.

## Declaration of competing interest

Herman is the owner of White Coat Artistry and does receive financial compensation for her artwork pieces through commission. Schukow is an ambassador for KiKo but he does not receive financial compensation for his position. Figures and artwork from Herman, Tatarian, El-Zaatari, and Sura are referenced in this review; however, they are done so for the purposes of expanding knowledge of PathArt and provide readers with sufficient examples and ideas for their own personal endeavors. The authors were not compensated for their image contributions. Bui does not receive financial compensation for her book *The Healing Art of Pathology* as proceeds fully go to charity.

## Figures and Tables

**figs1:**
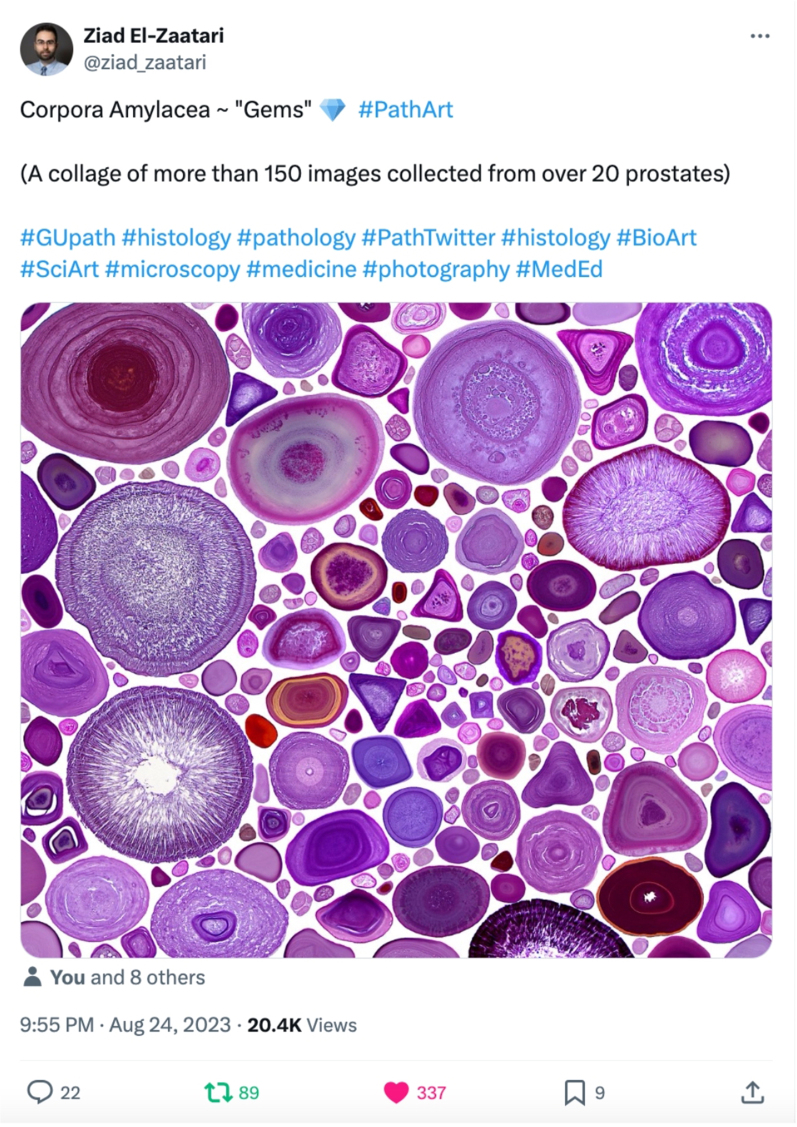


**figs2:**
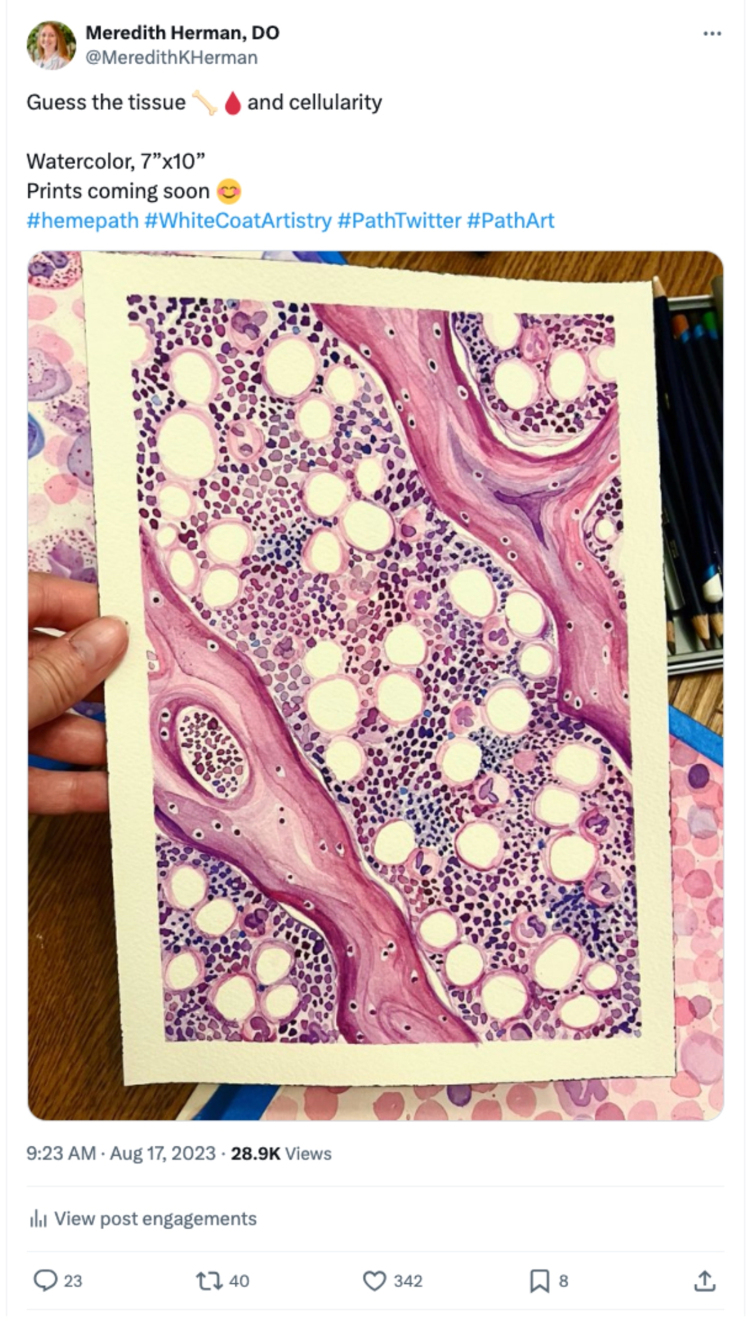


**figs3:**
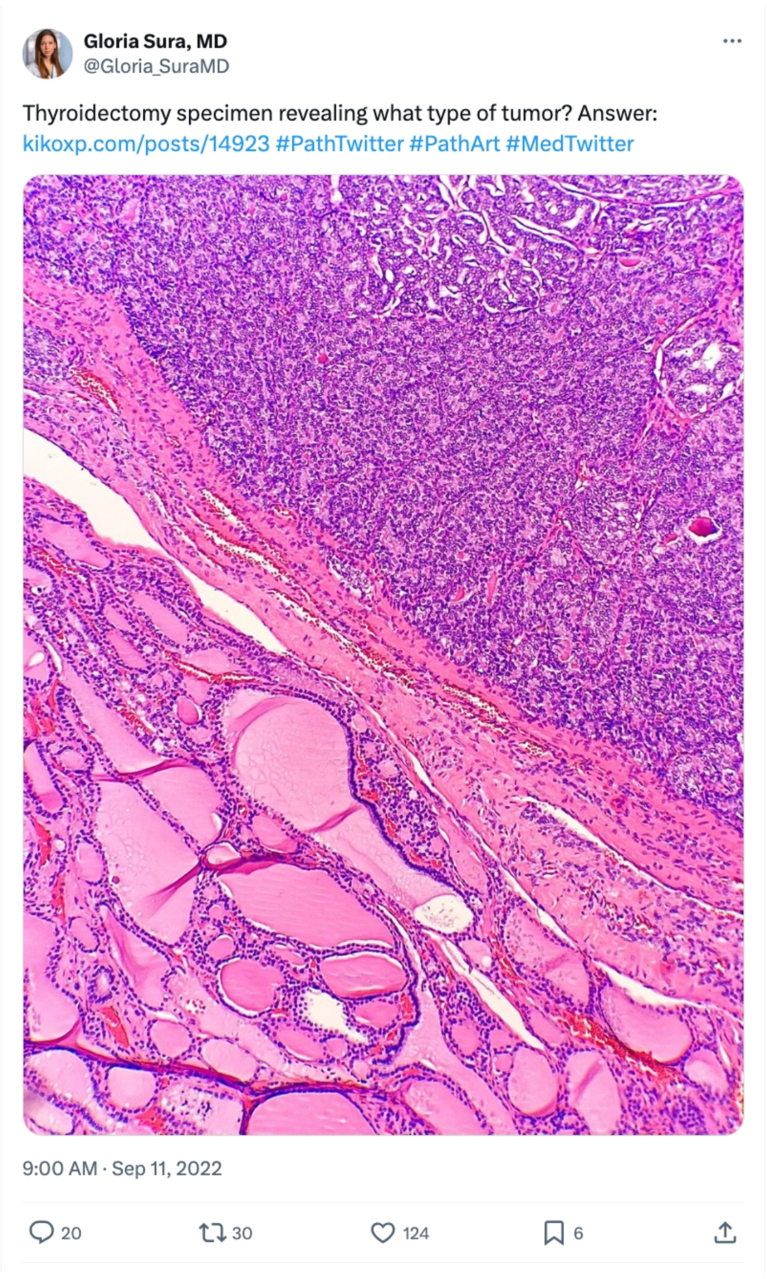


**figs4:**
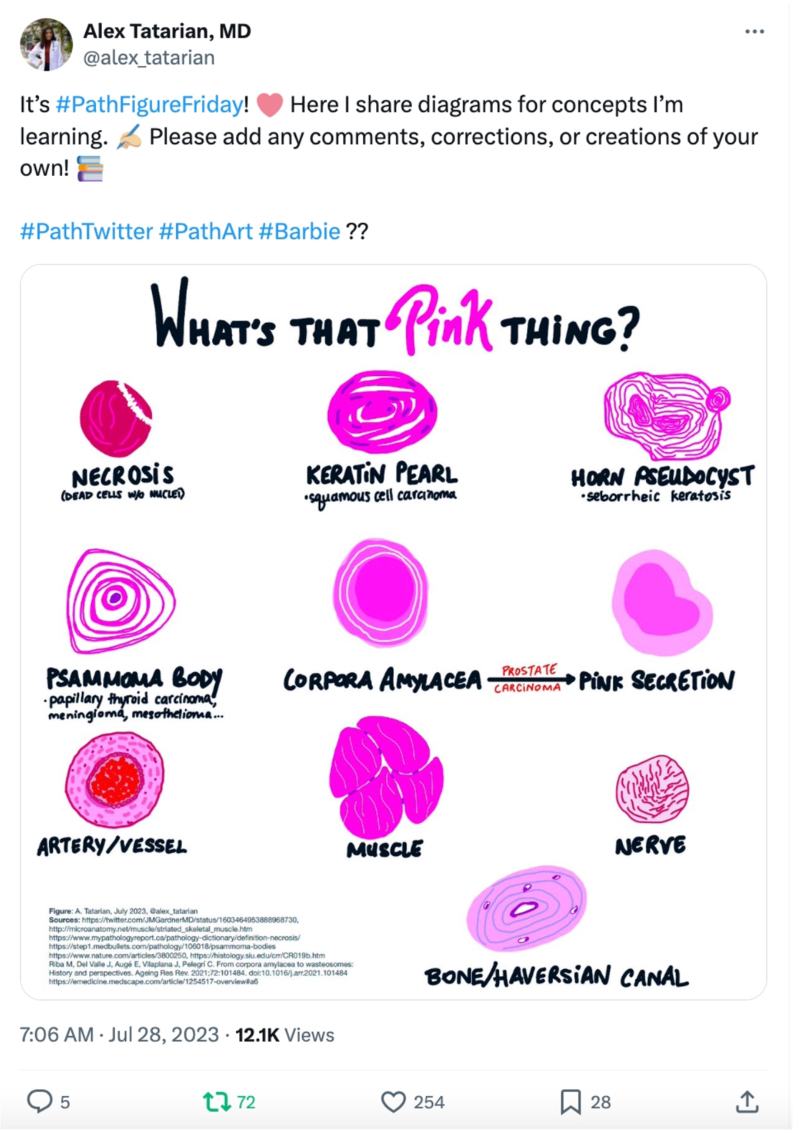


**figs5:**