# Supplementary material for: #PathArt: from glass slide to canvas; with a mission of enlightening the burdens of life
Source: Acad Pathol. 2025 Feb 3;12(1):100157. doi: 10.1016/j.acpath.2024.100157 (PMC11840207; doi:10.1016/j.acpath.2024.100157)
Supplement: Multimedia component 1 [file mmc1.pdf]

	AUTHOR	WEBSITE	INFLUENCE	MENTIONS
1	@Gloria_SuraMD	twitter.com	7,865	16
2	@ziad_zaatari	twitter.com	3,821	6
3	@AmyEngevik	twitter.com	2,345	4
4	JMGardnerMD	facebook.com	31,312	3
5	@belogrivceva_e	twitter.com	274	3
6	@iheartpath	twitter.com	2,413	2
7	@alex_tatarian	twitter.com	1,186	2
8	ihearthisto	facebook.com	38,097	2
9	@MadmusclesPlans	twitter.com	55,844	1
10	@karma_shopping	twitter.com	27,593	1
11	@mdlozanoe	twitter.com	5,204	1
12	@PeterKobalka	twitter.com	340	1
13	@MeredithKHerman	twitter.com	3,087	1
14	@dinesh_rakheja	twitter.com	1,028	1
15	@neuropathology	twitter.com	3,781	1
16	@PathDocBoston	twitter.com	7,306	1
17	@atman_ci	twitter.com	5,187	1
18	@VijayPatho	twitter.com	8,788	1
19	@kriyer68	twitter.com	13,463	1
20	@Drmerm	twitter.com	1,687	1
21	@rqbittenc	twitter.com	1,096	1
22	@pathologistmag	twitter.com	17,528	1
23	@NicoloVianini	twitter.com	64	1
24	@GunjanLShah	twitter.com	1,001	1
25	@tlabiano	twitter.com	5,572	1
26	@falarafa	twitter.com	382	1
27	aanpneuropathology	facebook.com	8,641	1
