# Supplementary material for: #PathArt: from glass slide to canvas; with a mission of enlightening the burdens of life
Source: Acad Pathol. 2025 Feb 3;12(1):100157. doi: 10.1016/j.acpath.2024.100157 (PMC11840207; doi:10.1016/j.acpath.2024.100157)
Supplement: Multimedia component 2 [file mmc2.docx]

**Supplemental Figure Legends**

**Supplemental Figure 1**: Twitter/X post of “Corpora Amylacea- Gems: Featuring a collage of more than 150 images collected from over 20 prostates.” Permission obtained from creator Ziad M. El-Zaatari (ZZ). Collected from Twitter search for #PathArt on June 17, 2023

**Supplemental Figure 2:** Twitter/X post of watercolor painting of bone marrow histology. Permission obtained from creator Meredith Herman (MH).

**Supplemental Figure 3:** Twitter/X post of thyroid histology stating “Thyroidectomy specimen revealing what type of tumor?” Permission obtained from creator Gloria Sura (GS).

**Supplemental Figure 4:** Twitter/X post of a diagram of digitally illustrating concepts in pathology, titled “What is that pink thing?” Permission obtained from creator Alexandra Tatarian (AT).

**Supplemental Figure 5:** Twitter/X post of a intestinal mucos for #MicroscopeMonday and #MucusMonday. Permission obtained from creator Amy Engevik (AE).

**Supplemental Figure 6:** List of authors ordered by the usage of #PathArt between Feb 7 to March 7, 2024. Data obtained from BrandMentions.com.^46, 47^
